# Comparative Analysis of the Intestinal Bacterial and RNA Viral Communities from Sentinel Birds Placed on Selected Broiler Chicken Farms

**DOI:** 10.1371/journal.pone.0117210

**Published:** 2015-01-30

**Authors:** J. Michael Day, Brian B. Oakley, Bruce S. Seal, Laszlo Zsak

**Affiliations:** 1 United States Department of Agriculture, Agricultural Research Service, Southeast Poultry Research Laboratory, Athens, GA, United States of America; 2 United States Department of Agriculture, Agricultural Research Service, Poultry Microbiological Safety Research Unit, Athens, GA, United States of America

## Abstract

There is a great deal of interest in characterizing the complex microbial communities in the poultry gut, and in understanding the effects of these dynamic communities on poultry performance, disease status, animal welfare, and microbes with human health significance. Investigations characterizing the poultry enteric virome have identified novel poultry viruses, but the roles these viruses play in disease and performance problems have yet to be fully characterized. The complex bacterial community present in the poultry gut influences gut development, immune status, and animal health, each of which can be an indicator of overall performance. The present metagenomic investigation was undertaken to provide insight into the colonization of specific pathogen free chickens by enteric microorganisms under field conditions and to compare the pre-contact intestinal microbiome with the altered microbiome following contact with poultry raised in the field. Analysis of the intestinal virome from contact birds (“sentinels”) placed on farms revealed colonization by members of the *Picornaviridae*, *Picobirnaviridae, Reoviridae*, and *Astroviridae* that were not present in pre-contact birds or present in proportionally lower numbers. Analysis of the sentinel gut bacterial community revealed an altered community in the post-contact birds, notably by members of the *Lachnospiracea/Clostridium* and *Lactobacillus* families and genera. Members of the avian enteric *Reoviridae* and *Astroviridae* have been well-characterized and have historically been implicated in poultry enteric disease; members of the *Picobirnaviridae* and *Picornaviridae* have only relatively recently been described in the poultry and avian gut, and their roles in the recognized disease syndromes and in poultry performance in general have not been determined. This metagenomic analysis has provided insight into the colonization of the poultry gut by enteric microbes circulating in commercial broiler flocks, and has identified enteric viruses and virus communities that warrant further study in order to understand their role(s) in avian gut health and disease.

## Introduction

Poultry performance is heavily influenced and ultimately dependent on the overall health and proper functioning of the avian gastrointestinal tract. The poultry gut is the site of nutrient absorption and can be a site for the introduction and proliferation of myriad microorganisms that may be pathogenic, beneficial, or benign. Poor gastrointestinal health can affect poultry performance in general—even in the absence of a recognized disease state—resulting in the failure of birds to reach well-established genetic potential. Poor performance due to poor gut health—coupled with non-specific enteric disease or microflora imbalance—results in poultry production problems such as poor feed conversions (a measure of the efficiency of the utilization of the nutrients in feed) and difficult management and animal health decisions such as the administration of antimicrobials. Poultry enteric problems are ongoing worldwide and result in significant economic loss to the poultry industry each year [1–5]. The poultry industry will benefit on many levels—disease prevention, improvements to animal welfare, and the realization of maximal performance—if the complex gut microbiome is better characterized and understood.

Clinically, enteric disease in poultry is marked by diarrhea, wet litter in poultry houses, decreased feed intake, dehydration, growth depression, and overall unevenness in the size of birds (lack of uniformity) [1,6,7]. Affected birds have pale, thin-walled intestines filled with undigested feed, and microscopically have blunted absorptive intestinal villi, indicating the inability of the intestines to absorb nutrients from feed; the non-specific enteric maladies in poultry have historically been referred to collectively as malabsorption syndromes, and affect poultry worldwide [7–10]. The recognized enteric disease syndromes in poultry—which include Runting-Stunting Syndrome (RSS) in young broiler chickens and Poult Enteritis Complex (PEC) in young turkeys (poults)—are true multifactorial diseases that have proven very difficult to reproduce experimentally in birds [8,11,12]. In fact, in order to reproduce the full complement of enteric signs observed in these syndromes, transmission studies have relied upon the administration of crude, uncharacterized intestinal homogenates prepared from affected birds, or by placing healthy birds on the litter previously used to rear affected flocks [13]. Many investigations have focused on individual intestinal viruses as possible etiologic agents in PEC and RSS [14–18], with many RNA viruses such as members of the *Astroviridae* and *Reoviridae* being implicated. To further complicate any investigation, suspect enteric viruses implicated in these syndromes are also often found in healthy poultry flocks, and pathogenic bacterial and even protozoal infections may be present in certain cases [1,9]. Much research has been completed characterizing individual viruses implicated in the poultry enteric disease syndromes, but recent investigations have moved necessarily toward community-based analyses of the gut microbiome. Recent metagenomic analyses of the poultry intestinal virome have revealed a number of uncharacterized viruses [19–21]; these viruses are truly novel and the role(s) they may play in the poultry enteric disease syndromes or in the overall gut health of poultry has not been determined. An investigation is necessary to determine the core viral constituency present in the poultry gut, and to identify viruses that warrant further characterization.

The complex bacterial community present in the poultry gut has received increased scrutiny as well, with 16S-based investigations providing an unprecedented look into the succession of poultry gut bacteria and the possible influence of that community on gut development, immune status, and health [22,23]. These prior 16S investigations have also identified specific bacterial species that show an association with performance deficiencies in poultry [4], or that may represent potential probiotic application for the poultry industry [5,24–26]. Depending on the section of the chicken gut examined, the numbers of bacteria in the gut can range from 10^9^ to 10^11^ per gram of gut contents, with many hundreds of distinct taxa typically present [27,28], and the members of the *Lactobacillus* genus dominating the ileum/jejunum while the *Clostridiaceae* dominate the cecum [5,23].

The present investigation utilized specific pathogen free (SPF) contact chickens (“sentinels”) placed on farms in order to provide a dynamic sample of bacteria and viruses capable of colonizing the avian intestinal tract through normal routes. This is in contrast to earlier investigations in which crude homogenates prepared in the laboratory from sampled poultry litter or intestinal contents are orally inoculated into experimental birds under isolated conditions. These sentinel birds represent an environmental sample that captures interactions with other birds in the flock and the environment of the house, farm, or yard in which the birds were placed. This investigation was undertaken to characterize both the bacterial and RNA virus communities present in chickens raised under SPF conditions both prior to and after placement on commercial and back yard chicken operations.

## Materials and Methods

### Placement of sentinels and receipt of intestinal samples

Commercial broiler chicken flocks—and one privately owned “back-yard” flock in the same geographical area—were selected based upon past problems with respiratory and enteric maladies. Five two-week old healthy contact birds (“sentinels”) were placed on the farms and allowed to remain in contact with the flocks for five days. The birds in the selected broiler flocks were each five weeks old at the time of contact placement. Contact birds originated from a commercial specific-pathogen free (SPF) flock; the non-contact, pre-placement SPF birds were maintained under SPF conditions and samples were labeled internally as “MSPF” to designate this fact; the contact birds were taken from the SPF flock and each given internal labels to represent placement on farms and to maintain the anonymity of commercial sources (GT, DM, TR, CAB, BY, VG, NG). Following the contact period, the contact birds were euthanized and the entire intestinal tract was removed; intestinal tracts from each set of five contact birds were pooled, frozen and shipped overnight to the Southeast Poultry Research Laboratory (SEPRL, ARS-USDA, Athens, GA). Pooled intestines were immediately processed via blending (Waring) into ~10% homogenates in sterile phosphate-buffered saline (PBS). Five pooled intestinal tracts from pre-contact SPF chickens were also received and homogenized separately from the contact birds’ intestines. No specific permits were required for the collection of the diagnostic samples by company veterinarians in the field, and care and handling of contact birds was in accordance with the USDA/ARS Southeast Poultry Research Laboratory (SEPRL) Institutional Animal Care and Use Committee (IACUC, A4298–01) guidelines and the SEPRL IACUC approved Animal Use Proposal FY2014–02.

### Gut homogenate preparation and viral RNA isolation

Pooled gut homogenates were clarified via centrifugation (2400 × G and 5500 × G; 15 min each, 4°C, SLA 1500 SuperLite rotor, Sorvall) and clarified supernatant was subjected to stepwise filtration through 0.8 micron and 0.45 micron cutoff syringe or bottle filters (Nalgene). The clarified, filtered homogenates were then spun at 41K for 4.5 hours at 4°C (Beckman Type 80 Ti, 113,000 × G). The resulting supernatant was removed and the virus pellet was re-suspended in sterile phosphate-buffered saline (PBS) via shaking at 4°C. The re-suspended pellet was aliquoted into cryogenic tubes and held at -80°C. DNAse and RNAse treatment of re-suspended viral particles was performed to minimized the amount of unencapsidated nucleic acid in the preparations: 450μl of the re-suspended pellet, 50μl 10X Turbo DNAse buffer (Ambion), 2.5μl Turbo DNAse (2Units/μl), 0.25μl RNAse A (20mg/ml, Invitrogen), 37°C for 1 hour. Following the nuclease treatment, total RNA was isolated from the viral particle suspension using a hybrid protocol with Trizol reagent and MagMax kit (Life Technologies) as described previously [29]. Multiple MagMax extractions were performed in concert using a KingFisher instrument (Thermo Scientific) to collect 250μl of viral RNA. Total viral RNA was precipitated via sodium acetate/ethanol precipitation, re-suspended in 50μl TE buffer, and stored at -80°C.

### Preparation and amplification of viral cDNA

cDNA first strand and second strand synthesis using random hexamers and oligo dT was performed on the total viral RNA using the SuperScript Choice System cDNA kit (Invitrogen). cDNA was ligated to the included *Eco*RI/*Not*I double-stranded oligonucleotide adapter and sephacryl-column purified to remove excess adapters to select for cDNA size (>500bp). Total viral cDNA was then subjected to a PCR amplification step using a single oligonucleotide (5’-CGG CCG CGT CGA C-3’) directed toward the ligated adapter sequence. The PCR reaction (50ul) was aliquoted into five equal reactions and subjected to thermal cycling (94C for 30s, 55C for 30s, 72C for 2 min for 35 cycles; final extension at 72C for 7 min); the aliquots were pooled following PCR and purified (Qiagen MinElute PCR cleanup kit). PCR amplicons were checked via agarose gel electrophoresis. Purified, amplified cDNA representing the total viral RNA was submitted directly for high-throughput sequencing. Prepared cDNA was barcoded and sequenced using the Ion Xpress Library Kit (Life Technologies) and the Ion Torrent Personal Genome Machine (PGM) platform.

### Enteric virome analysis

Raw nucleotide sequence for each pooled contact bird metagenome was uploaded to the MG-RAST metagenomics analysis server [30], and subjected to the stringent MG-RAST quality pipeline, which consisted of duplicate read inferred sequencing error estimation (DRISEE)—a method to estimate sequencing error that is independent of the high-throughput sequencing platform used [31]; a dereplication step to remove artificial duplicate reads (ADRs) [32]; and the generation of a nucleotide histogram to ensure approximately equal proportions of basecalls in these shotgun metagenomes. Viral taxon trees and the viral heatmap were generated using MG-RAST with annotation provided via a GenBank or M5 non-redundant (M5nr) [33] search with a maximum e-value cutoff of e^-5^, a minimum identity cutoff of 80%, and a minimum alignment overlap of 15nt. Viral metagenomes are publicly available at MG-RAST as project 2982 with MG-RAST metagenome identification numbers 4509871.3 and 4509873.3–4509879.3. Viral metagenome data is also available in the NCBI Sequence Read Archive (SRA) as a study with the accession number SRP033198.

### 16S rRNA bacteriome generation and analysis

PCR and 454 pyrosequencing of the V1–V3 regions of 16S rRNA genes were performed using tagged amplicon methods as previously described [34]. Briefly, sequences were de-multiplexed and preprocessed with the Galaxy toolkit [35] and our own custom tools [36]; additional quality controls per recent recommendations and standard protocols [37] were completed using Perl and Bioperl scripts to trim pyrosequencing tag sequences, screen for presence of the forward PCR primer sequence, and remove sequences with any ambiguous base calls. Based on expected amplicon sizes and frequency distributions of sequence lengths in v108 of the Silva reference database [38], sequences were further limited to a range of 325–425 bp. Putative chimeric sequences were identified with usearch [39] and ChimeraSlayer in mothur [40].

Taxonomic classification of sequences was performed with the Ribosomal Database Project (RDP) naïve Bayesian classifier [41] v2.6 and the EMBL taxonomy from v115 of the Silva project curated seed database using usearch with the global alignment option [39]. To assess phylotype richness and diversity independent of taxonomic classifications, sequences which passed all the screens described above were grouped into similarity clusters (operational taxonomic units; OTUs), using similarity cutoffs of 90%, 95%, and 97% with uclust [39]. The output from usearch provided the inputs for our own customized analysis pipeline to parse the clustering results and produce graphical and statistical summaries of the data for the desired sampling units using perl and R [42] as previously described [34,36]. Clustering of communities was performed using the CCA function of the vegan package [43] in R based on OTU and taxonomic classifications. For comparisons of bacterial genera significantly over- or under-represented in the contact birds, analysis was performed with the METASTATS package in R using taxonomic classifications from the RDP naïve Bayesian classifier. Differentially abundant taxa between the MSPF flock and each experimental flock were detected using the chi-square test in METASTATS [44]. Correlations of viral and bacterial taxa were done with frequency tables at various levels of taxonomic resolution using the R library corrplot. 16S bacterial metagenomes are publicly available at MG-RAST with metagenome identification numbers 4579001.3–4579008.3.

## Results and Discussion

### Clustering of 16S rRNA sequence data

**Fig 1:**
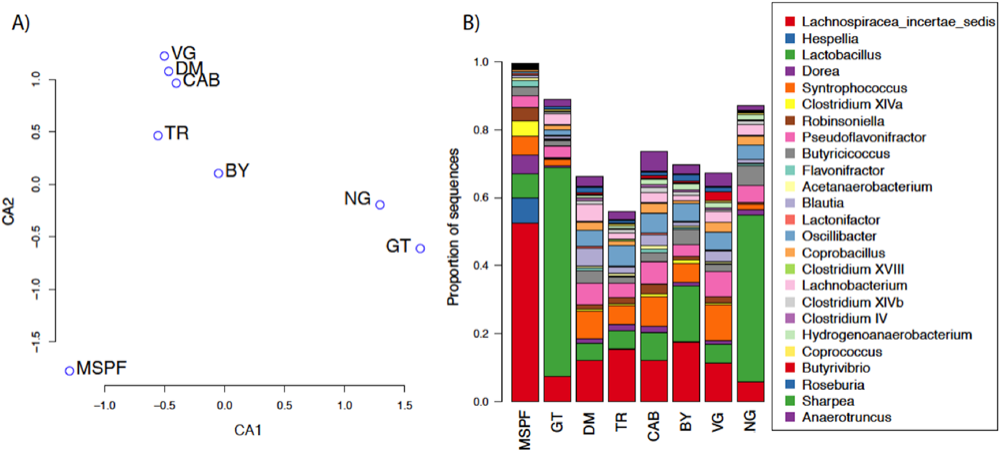
Clustering and Taxonomic Composition of Flocks. Clustering (A) and taxonomic composition (B) of the eight flocks according to 16S rRNA sequences of the intestinal microbial communities. Clustering of flocks was performed by canonical correspondence analysis and taxonomic classifications are based on the RDP naïve Bayesian classifier. Taxa with relative abundance <2.5% summed across all samples are excluded for clarity.

Clustering of 16S rRNA sequence data from each flock using CCA as described in the methods revealed the bacterial communities associated with the MSPF flock to be distinct from each of the experimental flocks (Fig. 1A). This result likely reflects the higher biosecurity conditions under which the MSPF flock was maintained compared to the commercial or back yard settings in which the sentinel birds were placed. Comparisons of the taxonomic composition of the communities showed the MSPF flock had a relatively high proportion of sequences classified by the RDP as Lachnospiracae incertae sedis and *Clostridium* XIVa (Fig. 1B). Both of these groups are members of the *Clostridiales* which are known to be the dominant taxonomic group in the ceca but are still somewhat poorly resolved taxonomically; the majority (>70%) of sequences classified as Lachnospiracae incertae sedis belonged to the genera *Blautia* or *Ruminoccus* according to the Silva taxonomy, while most (2/3) of the *Clostridium* XIVa sequences were classified simply in the genus *Clostridium* by comparison to the Silva database (v115). The similarities of the VG, DM, and CAB flocks (Fig. 1A) reflect their similar taxonomic profiles, particularly for the relative proportions of *Lactobacillus* and *Syntrophococcus* (Fig. 1B). Similarly, the communities of the NG and GT flocks were quite similar to one another (Fig. 1A), reflecting the relatively high proportion of *Lactobacillus* sequences (Fig. 1B). Compared to the MSPF pre-contact birds, each of the sentinel bird groups had genera specifically under- or over-represented in their intestines (Table 1). The GT, BY, and NG flocks each had significant over-representation by the *Blautia* genus compared to the pre-contact MSPF birds, while the CAB flock was the only flock with significantly more *Lactobacillus* spp. reads. The back yard (BY) flock was over-represented by members of the *Coprobacillus* genus, a group of bacteria that have only recently been detected and initially described in poultry [45]. The VG flock was the only flock with significant over-representation by members of the *Clostridium* XIVb cluster, and members of the *Robinsoniella* and *Lactonifactor* genera were under-represented in only the CAB and DM flocks, respectively. How these taxonomic differences affect bird health and nutrition remains unknown, but if the differences in community composition between the MSPF and experimental flocks do reflect differences in biosecurity measures, this result may have important implications for husbandry and management practices.

**Table 1:** Comparisons of Genera Significantly Over- or Under-Represented in Each Group of Sentinel Birds.

RDP_genus^1^	GT	DM	TR	CAB	BY	VG	NG
Acetanaerobacterium			0.95	1.32	0.85	0.99	
Blautia	1.28	0.79			1.50		1.42
Butyricicoccus			0.73	1.03		0.76	
Clostridium XlVb						2.36	
Coprobacillus					1.85		
Flavonifractor							
Lactobacillus				1.13			
Lactonifactor		0.87					
Pseudoflavonifractor	0.90		1.18		0.92		
Robinsoniella				0.81			

^1^ Proportions shown in **bold** indicate significant over-representation for a sentinel group relative to the pre-contact MSPF group, while ratios shown in *italics* indicate significant under-representation. Numbers indicate relative proportions for sentinel birds:pre-contact MSPF birds.

### Enteric RNA viral community analysis

**Fig 2:**
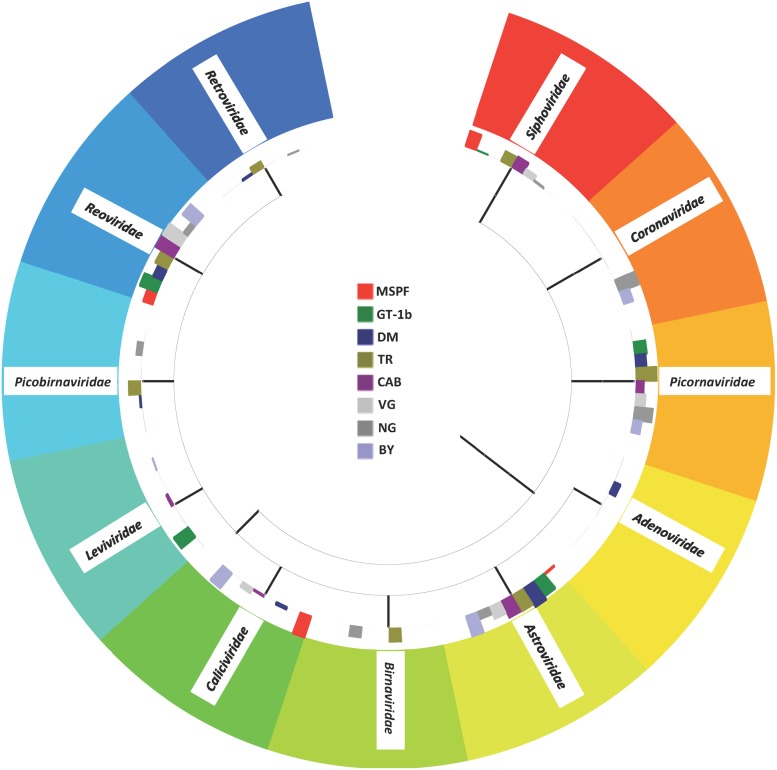
Virus Family Taxon Tree. Taxon tree representing the viral Families present in the gut viral metagenome for the pre-contact MSPF birds and the sentinel birds placed with each flock in the field. Search parameters as described in the text.

**Fig 3:**
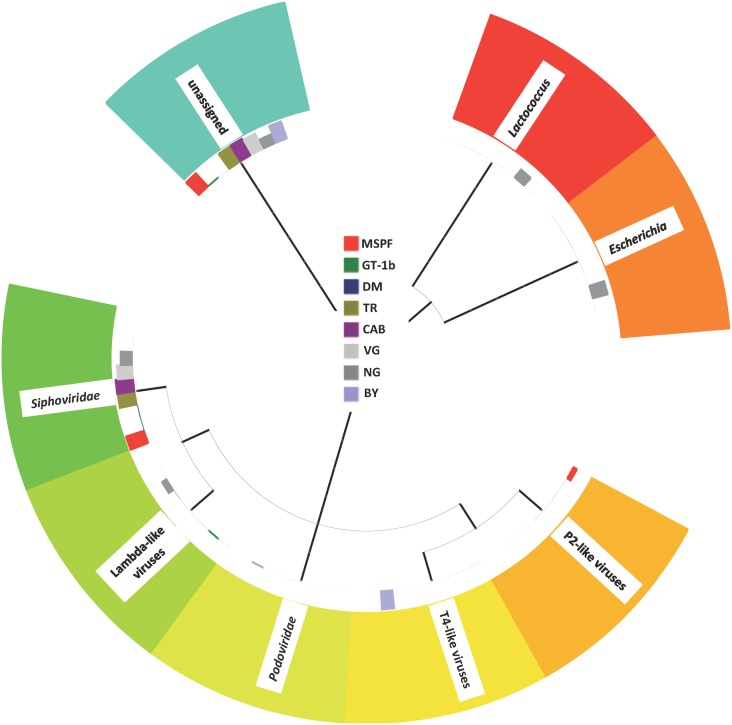
Phage Taxon Tree. Taxon tree representing the bacteriophage-associated viral reads present in the gut viral metagenome for the pre-contact MSPF birds and the sentinel birds placed with each flock in the field. Search parameters as described in the text.

**Fig 4:**
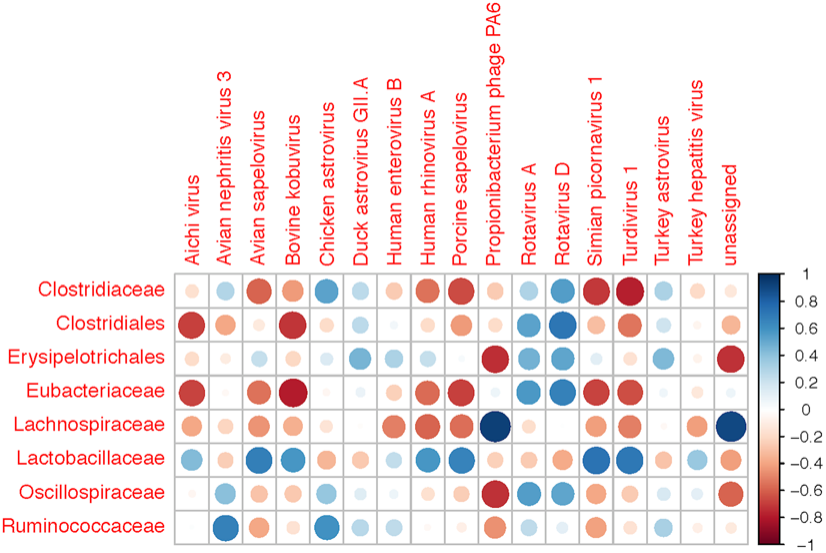
Correlation Matrix. Correlation matrices comparing viral taxa to bacteria at the Family level. The color and size of the cross-referenced circles corresponds to the level of correlation between groups. The legend indicates correlations (r values) ranging from-1 (strong negative correlation) to +1 (strong positive correlation).

The intestinal virome from the pre-contact MSPF birds and from the sentinels was marked by a number of RNA viruses that have historically been implicated in enteric disease and performance problems, and by a number of viruses only recently detected and described in poultry (Fig. 2). Interestingly, although the protocols used for preparation of the viral cDNA were specifically designed to mitigate the presence of DNA viral reads, homologies to several members of the *Siphoviridae* and the *Podoviridae* (tailed DNA phages, Fig. 3); further homologies were noted in certain sentinels to members of the T4- and P2-like viruses, which are members of the *Myoviridae* family of phages. The *Siphoviridae*, *Myoviridae*, and *Podoviridae* are members of the very large *Caudovirales* order and have been detected previously in a metagenomic analysis of the chicken gut microbiota [20,46]. Interestingly, the presence of the phage Propionibacterium phage PA6 (a member of the *Siphoviridae* family), correlated strongly with the presence of members of the Lachnospiraceae family of bacteria (Fig. 4), and with members of the Clostridium cluster XIVa (data not shown). Homologies to the avian *Adenoviridae* were also noted in the sentinels that were placed with the DM commercial flock, and specifically appear to be due to the presence of avian leukosis virus (ALV) in that flock. Members of the *Leviviridae* (+ssRNA phages) were also noted in some flocks, including the pre-contact MSPF birds; the *Leviviridae* have been noted previously in the enteric RNA viral metagenome from turkeys [19].

**Fig 5:**
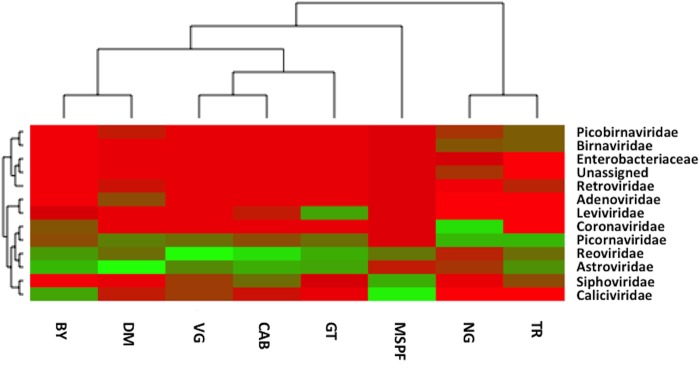
Viral Metagenome Heat Map. Heat map with dendogram (x-axis) representing the relationships between pre-contact MSPF birds and sentinel birds placed in contact with birds in the field. Family level viral comparisons are indicative of the viral abundance counts (y-axis) for each metagenome.

Of note in the pre-contact MSPF birds is that the majority (99.05%) of the viral sequence hits in the viral metagenome had homology to the members of the family *Caliciviridae* (S1 Fig.), with the remainder of viral reads belonging to the families *Reoviridae* (chicken rotavirus specifically) and *Astroviridae* (chicken astrovirus specifically). Astrovirus as well as rotavirus was detected in the sentinels placed on each of the commercial farms and the back yard farm as well (Fig. 2). The avian astroviruses and rotaviruses have been implicated in poultry enteric disease for decades, and a great deal of research has been completed on the prevalence and pathogenicity of these viruses [47–51]. The rotavirus infections correlated with the presence of several bacterial Families, including members of the *Clostridiaceae* and the *Eubacteriaceae* (Fig. 4), while the avian astroviruses—chicken astrovirus and avian nephritis virus specifically—correlated weakly with members of the *Clostridiaceae* and *Ruminococcaceae*. This could reflect the fact that the avian astroviruses are an almost ubiquitous constituent of the poultry gut [49,52]. Interestingly, the apparent *Calicivirus* infection appeared to have been completely cleared in the sentinel birds placed on the GT, TR, and NG commercial farms, and only comprised less than 0.05% of the viral hits from the sentinels placed on the additional commercial farms; 25.6% of the viral hits in the back yard (BY) flock had homology to the *Caliciviridae*. Members of the *Caliciviridae* were evident in an earlier metagenomic analysis of the turkey gut RNA virus community [19], and avian caliciviruses have been described previously in chickens with RSS and in otherwise healthy chickens and turkeys [53,54]. The majority of the hits observed in the MSPF and BY flocks showed homology to the calicivirus chicken/V0021/Bayern/2004. It is important to note that the SPF designation does not imply a chicken flock that is free of viral or bacterial infection; the designation means that specific (or specified) pathogens of concern are routinely assayed for and the biosecurity of the flock reflects the need to exclude these pathogens. SPF flocks have historically been assayed for as many as 31 potential pathogens [55], and it is likely that this list may have to be reexamined and perhaps updated as metagenomic analyses of poultry flocks becomes more common. Overall, the MSPF pre-contact birds had fewer viral reads in their viral metagenomes compared to the more complex viromes observed in sentinel birds that had spent five days in contact with flocks under field conditions (Fig. 5).

Two sets of sentinel birds, placed on flocks NG and BY, had viral reads with homology to the avian coronaviruses (order *Nidovirales*), with coronaviral reads comprising 77.2% of the total viral reads in the NG flock at the Order level (S2 Fig.). The coronaviral reads showed homology to avian infectious bronchitis virus (IBV) spike glycoprotein (S1 protein) and non-structural protein genes, and to the turkey enteric coronavirus polyprotein gene 1ab. Coronaviral reads comprised just a small percentage of the viral reads in the BY flock, with all reads having homology to the turkey enteric coronavirus polyprotein gene 1ab. It is interesting to speculate that the turkey enteric coronaviruses may be able to replicate in the chicken intestinal tract without causing enteric disease. IBV is primarily a respiratory disease in chickens, but it can affect other tissues and can replicate in the intestines without causing outward disease signs in birds [56–58].

Three sets of sentinels from flocks DM, TR, and NG had small numbers of viral reads with homology to the avian picobirnaviruses (PBVs). The PBVs were detected and described in a recent metagenomic analysis of the turkey gut viral metagenome [19], and have been described in chicken flocks as well [59,60]. As has been noted in all avian PBVs to date, the PBVs in the present analysis all appear to be members of the PBV genogroup I [59,61,62].

**Fig 6:**
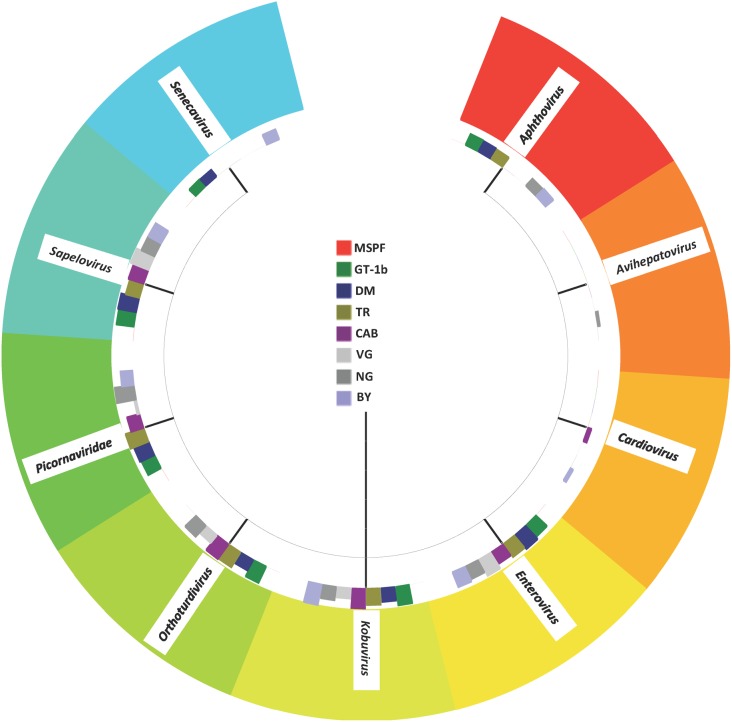
Taxon tree representing the *Picornaviridae* genera present in the gut viral metagenome for the pre-contact MSPF birds and the sentinel birds placed with each flock in the field.

Of particular interest in the present analysis is the observation that while the pre-contact MSPF birds had no avian picornaviral RNA present in their intestines, each of the sentinel groups had a significant number of viral reads with homologies to the members of the *Picornaviridae*. The picornaviral reads in the sentinel birds shared homology with multiple genera within the family *Picornaviridae* (Fig. 6), with many reads placed into the category “unclassified” as members of the Picornaviridae, as was noted in an earlier investigation of the turkey enteric virome [19]. Detailed investigations of the avian enteric picornaviruses suggest they may be widespread in poultry [21,63–65], and they have been described in wild birds (family *Turdidae*: robins and thrushes) from Hong Kong and given the name Turdiviruses as a result [66]. Turkey enteric picornaviruses have been described in birds in Hungary (*Gallivirus A* and *Avisivirus A*) [21,67]. As is the case with many of the poultry enteric viruses in turkeys and chickens, the role(s) the enteric avian enteric picornaviruses play in enteric disease and production loss is unclear at this time, although the present analysis supports earlier insight into the diversity of the picornaviruses in the chicken gut and the potential for the chicken to be a reservoir for the avian picornaviruses in general [65]. Of note is the general trend of negative correlation between members of the *Picornaviridae* Family (Aichi virus, Avian sapelovirus, Kobuvirus, and the avian Turdiviruses) and several bacterial Families, particularly the Clostridiaceae and the Eubacteriaceae. There was, however, a positive correlation between the members of the *Picornaviridae* and the *Lactobacillaceae* (Fig. 4).

## Conclusions

Previous surveys for enteric viruses in poultry have involved the use of multiple primer sets with specificity to previously described individual viruses, or multiplexed assays targeting known viruses [49,52,68,69]. The culture-independent approach presented here and in earlier investigations into the gut microbiome of agriculturally important animals provides a broader picture of the complex enteric microbial community. This investigation—which presents data on both the viral and bacterial communities in the poultry gut—has provided insight into the rapid colonization of the SPF chicken gut by numerous microbes in the commercial broiler and back yard flock environment. Further, although little is known about the possible causes of the negative and positive correlations noted among the viral and bacterial datasets analyzed here, this is an initial step toward quantifying the potential roles enteric viral infections might play in the disbacteriosis often noted in poultry with enteric disease and performance problems [1,4,9,12]. SPF sentinel birds are commonly employed to track the contact transmission of certain microbes, and in this investigation were used as an alternative sampling method to ensure that infective microbes were identified from the complex flock microbiota, and serves as proof-of-concept for collecting these complex samples in the future. In fact, as the protocols for high-throughput sequencing become more accessible and affordable, this sort of metagenomic/community-level analysis can be applied to diagnose animal disease through the identification of disease- or low performance-associated microbes or genes that are not present in the core microbiome of healthy pre-contact (i.e., SPF) animals.

## Supporting Information

S1 Fig Viral hit percentages for each metagenome, Family level. MG-RAST accession numbers correspond to flock designations in the text: 4509879.3 = MSPF; 4509871.3 = GT; 4509873.3 = DM; 4509874.3 = TR; 4509875.3 = CAB; 4509876.3 = VG; 4509877.3 = NG; and 4509878.3 = BY.
(TIFF)

S2 Fig Viral hit percentages for each metagenome, Order level. MG-RAST accession numbers correspond to flock designations in the text: 4509879.3 = MSPF; 4509871.3 = GT; 4509873.3 = DM; 4509874.3 = TR; 4509875.3 = CAB; 4509876.3 = VG; 4509877.3 = NG; and 4509878.3 = BY.
(TIFF)
