# Supplementary figures and images for: Comparative Analysis of the Intestinal Bacterial and RNA Viral Communities from Sentinel Birds Placed on Selected Broiler Chicken Farms
Source: PLoS One. 2015 Jan 30;10(1):e0117210. doi: 10.1371/journal.pone.0117210 (PMC4311960; doi:10.1371/journal.pone.0117210)

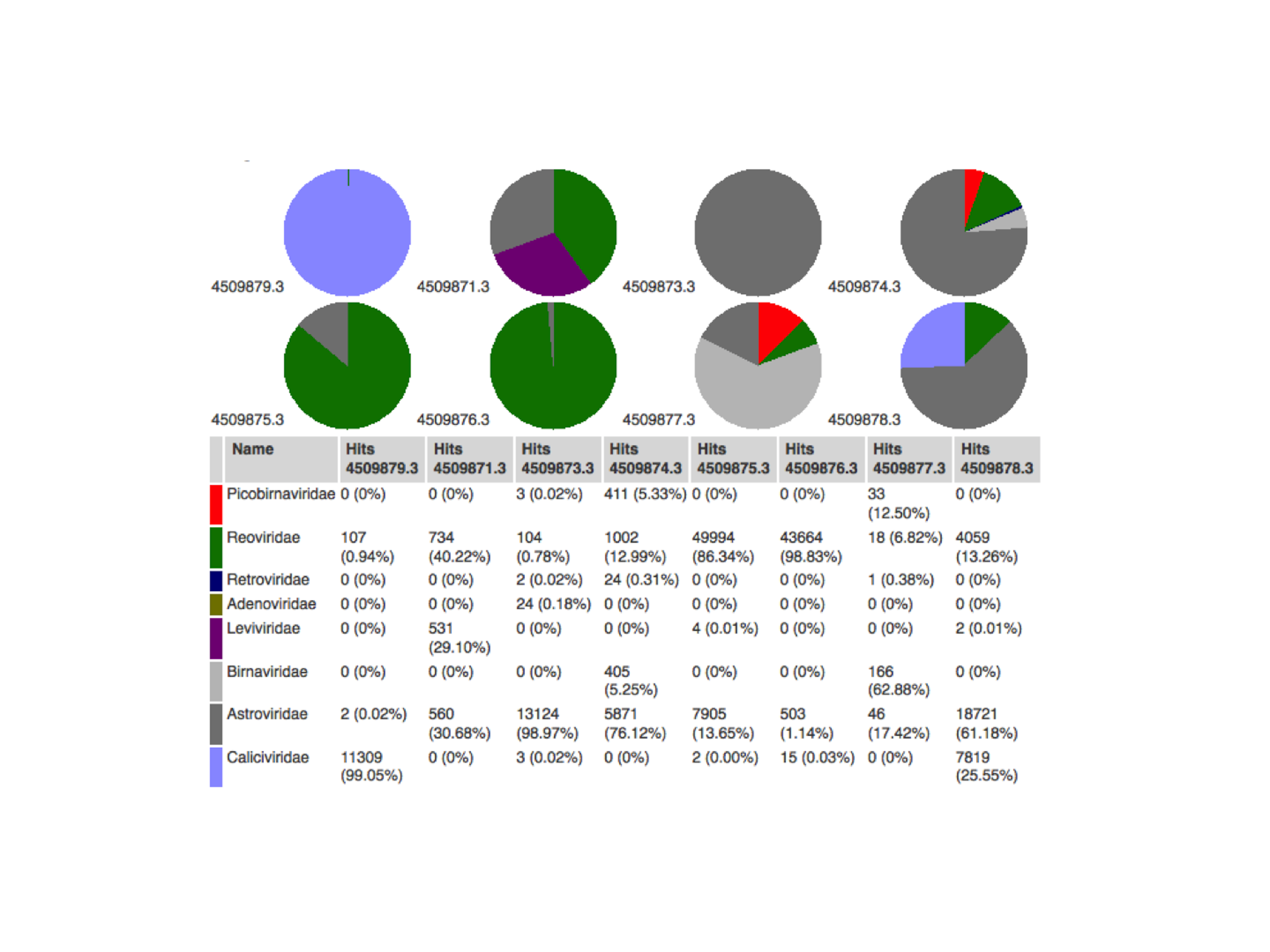

Supplement: S1 Fig — MG-RAST accession numbers correspond to flock designations in the text: 4509879.3 = MSPF; 4509871.3 = GT; 4509873.3 = DM; 4509874.3 = TR; 4509875.3 = CAB; 4509876.3 = VG; 4509877.3 = NG; and 4509878.3 = BY. (TIFF) [file pone.0117210.s001.tiff]

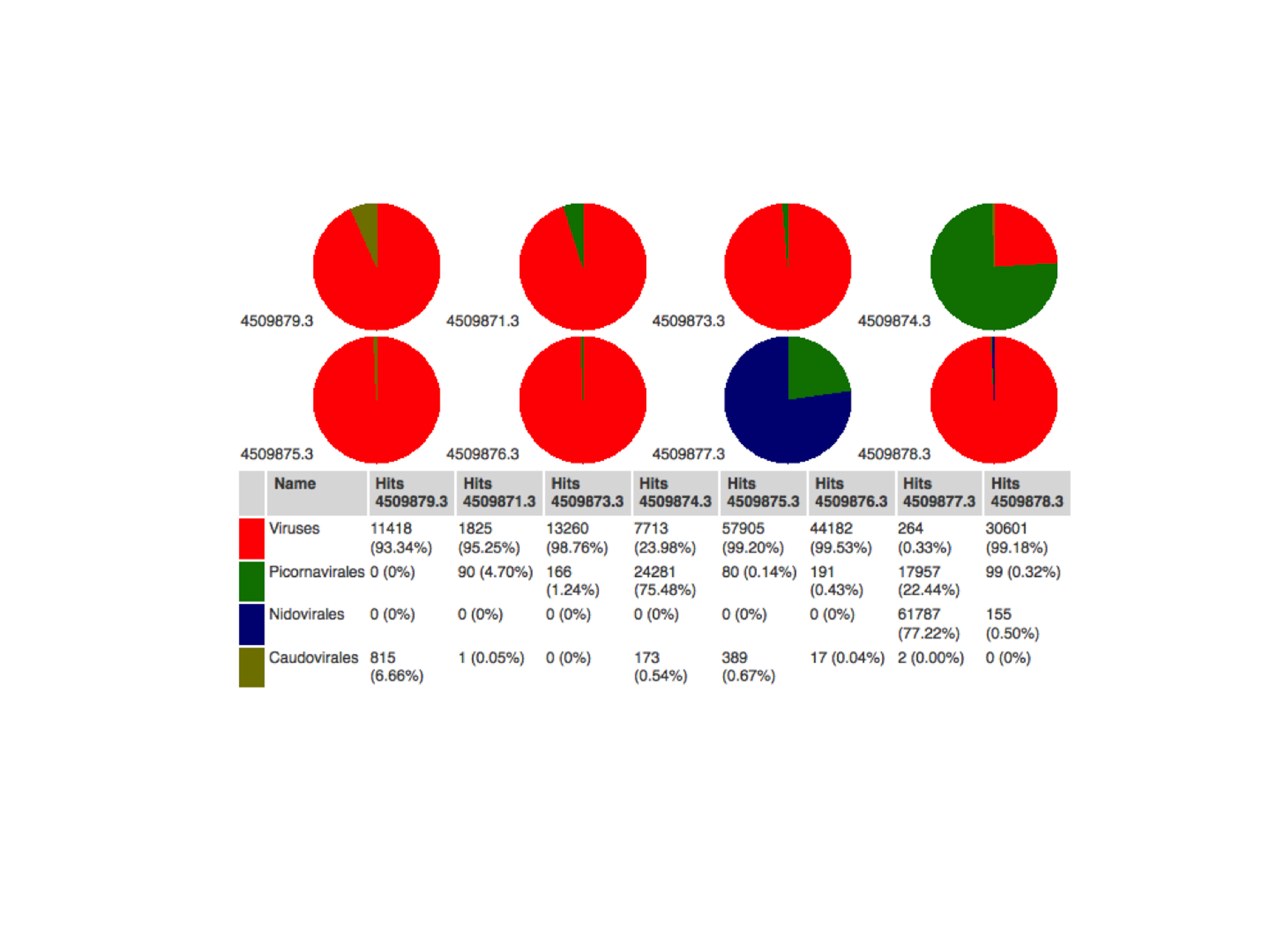

Supplement: S2 Fig — MG-RAST accession numbers correspond to flock designations in the text: 4509879.3 = MSPF; 4509871.3 = GT; 4509873.3 = DM; 4509874.3 = TR; 4509875.3 = CAB; 4509876.3 = VG; 4509877.3 = NG; and 4509878.3 = BY. (TIFF) [file pone.0117210.s002.tiff]
